# Effects of citrus pulp, fish by-product and *Bacillus subtilis* fermentation biomass on growth performance, nutrient digestibility, and fecal microflora of weanling pigs

**DOI:** 10.1186/2055-0391-56-10

**Published:** 2014-07-28

**Authors:** Hyun Suk Noh, Santosh Laxman Ingale, Su Hyup Lee, Kwang Hyun Kim, Ill Kyong Kwon, Young Hwa Kim, Byung Jo Chae

**Affiliations:** Department of Animal Resources Science, College of Animal Life Sciences, Kangwon National University, Chuncheon, 200-701 South Korea; Department of Animal Products and Food Science, College of Animal Life Sciences, Kangwon National University, Chuncheon, 200-701 South Korea; Department of Animal Resources Development, Swine Science Division, National Institute of Animal Science, RDA, Suwon, South Korea

**Keywords:** Citrus pulp, *Bacillus subtilis*, Performance, Fecal microflora, Weanling pigs

## Abstract

An experiment was conducted to investigate the effects of dietary supplementation with citrus pulp, fish by-product, and *Bacillus subtilis* fermentation biomass on the growth performance, apparent total tract digestibility (ATTD) of nutrients, and fecal microflora of weanling pigs. A total of 180 weaned piglets (Landrace × Yorkshire × Duroc) were randomly allotted to three treatments on the basis of body weight (BW). There were six replicate pens in each treatment with 10 piglets per pen. Dietary treatments were corn-soybean meal-based basal diet supplemented with 0 (control), 2.5, and 5.0% citrus pulp, fish by-product, and *B. subtilis* fermentation biomass. The isocaloric and isoproteineous experimental diets were fed in mash form in two phases (d 0 ~ 14, phase I and d 15 ~ 28, phase II). Dietary treatments had significant linear effects on gain to feed ratio (G:F) in all periods, whereas significant linear effects on ATTD of dry matter (DM), gross energy (GE), and ash were only observed in phase I. Piglets fed diet supplemented with 5.0% citrus pulp, fish by-product, and *B. subtilis* fermentation biomass showed greater (*p* < 0.05) G:F (phase I, phase II, and overall) as well as ATTD of DM, GE, and ash (phase I) than pigs fed control diet. Dietary treatments also had significant linear effects on total anaerobic bacteria populations by d 14 and 28. In addition, piglets fed diet supplemented with 5.0% citrus pulp, fish by-product and *B. subtilis* fermentation biomass showed greater (*p* < 0.05) fecal total anaerobic bacteria populations (d 14 and 28) than pigs fed control diet. Dietary treatments had no significant effects (linear or quadratic) on average daily gain (ADG), average dial feed intake (ADFI; phase I, phase II, and overall), or fecal populations of *Bifidobacterium* spp., *Clostridium* spp., and coliforms (d 14 and 28). These results indicate that dietary supplementation with 5.0% citrus pulp, fish by-product, and *B. subtilis* fermentation biomass has the potential to improve the feed efficiency, nutrient digestibility, and fecal microflora of weanling pigs.

## Background

Worldwide production of citrus fruits approaches 90 million tons per year [1]. Most of these fruits are squeezed to juice and by-products, including peels, segment membranes, and other parts, which are considered as citrus juice waste or pulp [2]. Due to their high processing cost, most citrus juice industry by-products are dumped into the ocean, leading to environmental pollution [3]. Recent approaches advocating the use of citrus juice waste have focused on reduction of its moisture contents and its use as an animal feed [4]. Dried citrus pulp contains relatively large amounts of pectins [5] and soluble carbohydrates [6]. Further, several health-promoting bioactive compounds such as limonene, hesperidin, naringin, quercetin, and bioflavonoids have been identified in citrus pulp [3, 7–9]. Contreras Esquivel et al. [10] and Sen et al. [11] reported that citrus juice industry by-products have the necessary characteristics required for substrates of probiotic growth during fermentation.

Among probiotic microbes, *Bacillus* spp. are well known for their ability to produce pectinase using pectin in citrus peel as the sole carbon source [12, 13]. Previous have reported that citrus juice waste can be used as a substrate for the growth of *B. subtilis*, and the resulting fermentation biomass has potential for improving the performance, intestinal morphology, and cecal microflora of broilers and weanling pigs [11, 14]. Therefore, the objective of the present study was to investigate the effects of dietary supplementation with citrus pulp, fish by-product, and *B. subtilis* fermentation biomass on growth performance, apparent total tract digestibility (ATTD) of nutrients, and fecal microflora in weanling of pigs.

## Methods

The protocol for the present experiment was approved by the Institutional Animal Care and Use Committee of Kangwon National University, Chuncheon, Republic of Korea.

### Preparation of fermentation biomass

The *B. subtilis* used in the present study was isolated and characterized by Yoo et al. [3] and maintained in the laboratory at −80°C as stock culture. Culture broth medium consisting of 6% corn steep liquor, 4% molasses, 0.3% yeast extract, 0.5% KH_2_PO_4_, and 0.25% K_2_HPO_4_ in distilled water was prepared and autoclaved before being used. Stock culture (2 mL) was then added to 2 L of autoclaved culture broth and incubated at 37°C at pH 7.0 for 48 h.

The *B. subtilis* grown on culture broth medium was used as a starter to produce fermentation biomass. Dried citrus pulp and fish by-product with 30% moisture was used as the sole substrate. The substrate was inoculated with 2 L of starter per 10 kg of substrate and fermented for 7 d at 32°C and pH 7.0. After 7 d, the fermentation biomass was dried in a forced-air drying oven at 40°C for 72 h.

### Animals and experimental design

A total of 180 weaned piglets (Landrace × Yorkshire × Duroc) were randomly allotted to three treatments on the basis of initial body weight (BW). There were six replicate pens in each treatment with 10 piglets per pen. All piglets were clinically healthy at the start of the trial and originated from 20 sows in their third parity. Dietary treatments were corn-soybean meal-based basal diet supplemented with 0 (control), 2.5, and 5.0% citrus pulp, fish by-product, and *B. subtilis* fermentation biomass. The citrus pulp, fish by-product and *B. subtilis* fermentation biomass were added to the weanling pig diets by replacing equal volumes of fish meal. The isocaloric and isoproteineous experimental diets were fed in mash form in two phases (d 0 ~ 14, phase I and d 15 ~ 28, phase II). Diets for phase I were formulated to contain 3,400 kcal/kg metabolizable energy (ME), 21.0% crude protein (CP), and 1.6% lysine (Table 1), whereas diets for phase II were formulated to contain 3,360 kcal/kg ME, 20.0% CP, and 1.4% lysine (Table 2). All diets met or exceeded the nutrient requirements recommended by NRC [15].

**Table 1:** **Ingredient and chemical compositions of experimental diets (d 0 ~ 14; as-fed basis)**

	Citrus pulp, fish by-product, and *Bacillus subtilis*fermentation biomass, %		
	0	2.5	5
Ingredients, %			
Corn	45.38	42.36	39.34
Whey powder	15.38	15.38	15.38
Deh-SBM	24.88	27.08	29.29
Soy oil	3.00	3.00	3.00
Fish meal	5.0	2.5	0.0
L-lysine (78%)	0.50	0.51	0.52
DL-Methionine (100%)	0.12	0.14	0.15
Choline-chloride (50%)	0.07	0.07	0.07
MCP	0.50	0.79	1.08
Limestone	0.67	0.73	0.78
Salt	0.20	0.20	0.20
Mineral premix^1^	0.30	0.30	0.30
Vitamin premix^2^	0.30	0.30	0.30
ZnO	0.34	0.34	0.34
Sucrose	3.36	3.80	4.25
Citrus pulp, fish by-product, and *Bacillus subtilis* fermentation biomass	0.0	2.50	5.00
Calculated chemical composition^3^			
ME, kcal/kg	3,400	3,400	3,400
CP, %	21.00	21.00	21.00
Ca, %	0.80	0.80	0.80
Av. P, %	0.40	0.40	0.40
Lys, %	1.60	1.60	1.60
Met + Cys, %	0.80	0.80	0.80

^1^Supplied per kilogram of diet: 45 mg Fe, 0.25 mg Co, 50 mg Cu, 15 mg Mn, 25 mg Zn, 0.35 mg I, 0.13 mg Se.

^2^Supplied per kilogram of diet: 16,000 IU vitamin A, 3,000 IU vitamin D3, 40 IU vitamin E, 5.0 mg vitamin K3, 5.0 mg vitamin B1, 20 mg vitamin B2, 4 mg vitamin B6, 0.08 mg vitamin B12, 40 mg pantothenic acid, 75 mg niacin, 0.15 mg biotin, 0.65 mg folic acid, 12 mg antioxidant.

^3^Based on NRC [15] values.

**Table 2:** **Ingredient and chemical compositions of experimental diets (d 15 ~ 28; as-fed basis)**

	Citrus pulp, fish by-product, and *Bacillus subtilis*fermentation biomass, %		
	0	2.5	5
Ingredients, %			
Corn	61.55	59.05	56.55
Whey powder	5.00	5.00	5.00
Deh-SBM	23.11	25.23	27.35
Soy oil	2.21	2.23	2.25
Fish meal (60%)	5.00	2.50	-
_L_-lysine (78%)	0.38	0.39	0.40
_DL_-Methionine (100%)	0.05	0.06	0.07
MCP	0.71	1.00	1.29
Limestone	0.75	0.80	0.85
Salt	0.30	0.30	0.30
Mineral premix^1^	0.30	0.30	0.30
Vitamin premix^2^	0.30	0.30	0.30
ZnO	0.34	0.34	0.34
Citrus pulp, fish by-product, and *Bacillus subtilis* fermentation biomass	0.0	2.50	5.00
Calculated chemical composition^3^			
ME, kcal/kg	3,360	3,360	3,360
CP, %	20.00	20.00	20.00
Ca, %	0.78	0.78	0.78
Av. P, %	0.37	0.37	0.37
Lys, %	1.40	1.40	1.40
Met + Cys, %	0.72	0.72	0.72

^1^Supplied per kilogram of diet: 45 mg Fe, 0.25 mg Co, 50 mg Cu, 15 mg Mn, 25 mg Zn, 0.35 mg I, 0.13 mg Se.

^2^Supplied per kilogram of diet: 16,000 IU vitamin A, 3,000 IU vitamin D_3_, 40 IU vitamin E, 5.0 mg vitamin K_3_, 5.0 mg vitamin B_1_, 20 mg vitamin B_2_, 4 mg vitamin B_6_, 0.08 mg vitamin B_12_, 40 mg pantothenic acid, 75 mg niacin, 0.15 mg biotin, 0.65 mg folic acid, 12 mg antioxidant.

^3^Based on NRC [15] values.

The experiment was conducted at the Kangwon National University farm facility. The piglets were housed in partially slatted concrete floor pens (2.8 × 5.0 m). The temperature in the barn was 30°C at the beginning of the experiment and was slowly reduced to 25°C on d 8, after which it was kept constant until the end of the trial. The humidity ranged between 60 and 70%. Each pen was equipped with an infrared heating lamp, self-feeder, and low-pressure nipple drinker to allow *ad libitum* access to feed and water.

### Experimental procedure and sampling

The pigs were individually weighed at the beginning of the trial as well as on d 14 and 28 of the experiment. Feed that was not consumed was weighed at the end of each phase, and consumption was calculated for phase I (d 0 to 14), phase II (d 15 to 28), and for the overall study period (d 0 to 28). As feed wastage was considered minimal, feed disappearance was determined to be a reliable estimate of feed consumption. Feed consumption was calculated at the end of each phase, and average daily feed intake (ADFI) and gain to feed ratio (G:F) were calculated. Average daily gain (ADG) and ADFI were calculated by dividing total pen weight gain and total pen feed consumption by the number of days. The G:F for each pen was calculated by dividing the ADG by the ADFI. To evaluate the effects of dietary treatments on the apparent total tract digestibility (ATTD) of energy and nutrients, 2.5 g kg^−1^ of chromium (as an inert, indigestible indicator) was included in the diets from d 8 to 14 (phase I) or d 22 to 28 (phase II). Fecal grab samples (100 gm/d per pen) were collected from each pen during the last 3 d of each phase to determine the ATTD of nutrients. Fecal samples from each pen were pooled, dried in a forced air-drying oven at 60°C for 72 h, and ground in a Wiley laboratory mill (Thomas Model 4 Wiley® Mill, Thomas scientific, Swedesboro, NJ, USA) using a 1-mm screen. Additionally, fresh fecal samples were collected from two pigs from each pen on d 14 and 28 and then measured for fecal bacterial counts. The samples collected for microbial analysis were immediately placed on ice (2–3 h) and transported to the laboratory for further analysis on the same day.

### Chemical and microbial analyses

Experimental diets and excreta samples were analyzed in triplicate for dry matter (DM, method 930.15; [16]), crude protein (CP, method 990.03; [16]), ash (method 942.05; [16]), calcium, and phosphorus (method 985.01; [16]). Gross energy was measured by a bomb calorimeter (Model 1261, Parr Instrument Co., Moline, IL), and chromium concentrations of experimental diets and excreta samples were determined with an automated spectrophotometer (Jasco V-650, Jasco Corp., Tokyo, Japan) according to the procedure of Fenton and Fenton [17]. The ATTD (%) of nutrients was calculated by the following formula:


Where

N_f_ = nutrient concentration in feces (%)

N_d_ = nutrient concentration in diet (%)

C_f_ = chromium concentration in feces (%)

C_d_ = chromium concentration in diet (%)

The microbiological assay of excreta was carried out by the procedure suggested by Choi et al. [18]. The microbial groups enumerated were total anaerobic bacteria (TAB; plate count agar, Difco Laboratories, Detroit, MI, USA), *Bifidobacterium* spp. (MRS agar), coliforms (violet red bile agar, Difco Laboratories, Detroit, MI, USA), and *Clostridium* spp. (Tryptose sulphite cycloserine agar, Oxoid, Hampshire, UK). The anaerobic conditions during the TAB and *Clostridium* spp. assays were created by using the gas-pak anaerobic system (BBL, No. 260678, Difco, Detroit, MI, USA).

### Statistical analysis

Data generated in the present study were subjected to statistical analysis using the GLM procedure of SAS (SAS Inst. Inc., Cary, NC) in a randomized complete block design. When significant difference were identified among treatment means, they were separated using Tukey’s Honestly Significant Difference test. The linear and quadratic contrasts were used to compare effects of increasing levels of citrus pulp, fish by-product, and *B. subtilis* fermentation biomass (0. 2.5 and 5.0% of basal diet). The pen was used as the experimental unit for the analysis of all parameters. Probability values of ≤0.05 were considered significant.

## Results

### Growth performance

Dietary treatments had significant linear effects on gain to feed ratio (G:F) in all periods (Table 3). Moreover, piglets fed diet supplemented with 5.0% citrus pulp, fish by-product and *B. subtilis* fermentation biomass showed greater (*p* < 0.05) G:F than piglets fed control diet. The G:F of piglets fed diet supplemented with 2.5% citrus pulp and *B. subtilis* fermentation biomass was not different (*p* > 0.05) from that of piglets fed control or 5.0% citrus pulp, fish by-product, and *B. subtilis* fermentation biomass. Dietary treatments had no effects (linear or quadratic; *p* > 0.05) on the ADG or ADFI of piglets in all periods.

**Table 3:** **Effect of dietary inclusion of citrus pulp, fish by-product and** ***Bacillus subtilis*** **fermentation biomass on growth performance of weanling pigs** ^**1**^

Item	Citrus pulp, fish by-product, and *Bacillus subtilis*fermentation biomass, %			SEM^2^	P-values^3^	
	0	2.5	5.0		Linear	Quadratic
Phase I (d 0 ∼ 14)						
ADG, g	441	460	474	14.86	0.145	0.871
ADFI, g	721	706	689	8.51	0.053	0.937
G:F, g/kg	613^b^	652^ab^	688^a^	19.96	0.018	0.960
Phase II (d 15 ∼ 28)						
ADG, g	515	540	576	16.80	0.057	0.688
ADFI, g	1,087	1,129	1,123	13.36	0.163	0.686
G:F, g/kg	474^b^	478^ab^	512^a^	9.06	0.018	0.199
Overall (d 0 ∼ 28)						
ADG, g	478	500	525	11.40	0.250	0.944
ADFI, g	904	917	906	13.74	0.058	0.747
G:F, g/kg	529^b^	545^ab^	579^a^	11.53	0.024	0.545

^ab^Values with different superscripts of the row significantly differ (p < 0.05).

^1^Data represent means based on 6 replicates per treatment.

^2^Standard error of means.

^3^Linear and quadratic effects of increasing citrus pulp and *Bacillus subtilis* fermentation biomass in the diet.

### Apparent total tract digestibility

Significant linear effects on ATTD of DM, GE, and ash were only observed in phase I (Table 4). In addition, in phase I, piglets fed diet supplemented with 5.0% citrus pulp and *B. subtilis* fermentation biomass showed greater (*p* < 0.05) ATTD of DM, GE, and ash than piglets fed control diet. However, ATTD of DM, GE, and ash of piglets fed diet supplemented with 2.5% citrus pulp, fish by-product, and *B. subtilis* fermentation biomass was not different (*p* > 0.05) than those of pigs fed control and 5.0% citrus pulp, fish by-product, and *B. subtilis* fermentation biomass. During phase II, dietary treatments had no effects (linear or quadratic; *p* > 0.05) on ATTD of nutrients.

**Table 4:** **Effect of dietary inclusion of citrus pulp, fish by-product, and** ***Bacillus subtilis*** **fermentation biomass on apparent total tract digestibility (%) of nutrients in weanling pigs** ^**1**^

Item	Citrus pulp, fish by-product, and *Bacillus subtilis*fermentation biomass, %			SEM^2^	P-values^3^	
	0	2.5	5.0		Linear	Quadratic
Phase I (d 8–14)						
DM	84.63^b^	84.99^ab^	85.49^a^	0.15	0.003	0.714
GE	83.56^b^	83.89^ab^	84.19^a^	0.12	0.005	0.890
CP	79.66	79.93	80.21	0.275	0.098	0.906
Ash	60.02^b^	60.76^ab^	61.72^a^	0.26	0.005	0.461
Ca	56.48	55.52	54.99	1.77	0.548	0.851
P	45.58	45.22	45.04	1.03	0.690	0.592
Phase II (d 22–28)						
DM	81.87	82.04	82.77	0.35	0.106	0.535
GE	82.14	82.67	83.34	0.47	0.101	0.909
CP	75.95	76.78	77.39	0.61	0.127	0.885
Ash	54.98	55.34	55.57	0.75	0.595	0.947
Ca	52.08	52.34	52.53	1.38	0.821	0.983
P	46.96	47.57	48.67	1.52	0.448	0.894

^ab^Values with different superscripts of the row significantly differ (p < 0.05).

^1^Data represent means based on 6 replicates per treatment.

^2^Standard error of means.

^3^Linear and quadratic effects of increasing citrus pulp and *Bacillus subtilis* fermentation biomass in the diet.

### Fecal microflora

Dietary treatments also had significant linear effects on total anaerobic bacteria populations by d 14 and 28 (Table 5). Moreover, piglets fed diet supplemented with 5.0% citrus pulp, fish byproduct, and *B. subtilis* fermentation biomass showed greater (*p* < 0.05) fecal total anaerobic bacteria populations than piglets fed control diet. However, total anaerobic bacteria populations of piglets fed diet supplemented with 2.5% citrus pulp, fish by-product, and *B. subtilis* fermentation biomass were not different (*p* > 0.05) than those of piglets fed control or 5.0% citrus pulp and *B. subtilis* fermentation biomass. Dietary treatments had no effects (linear or quadratic; *p* > 0.05) on populations of fecal *Bifidobacterium* spp., *Clostridium* spp., and coliform (d 14 and 28).

**Table 5:** **Effect of dietary inclusion of citrus pulp, fish by-product, and** ***Bacillus subtilis*** **fermentation biomass on fecal microflora (Log**_**10**_ **cfu/g) of weanling pigs** ^**1**^

Item	Citrus pulp, fish by-product, and *Bacillus subtilis*fermentation biomass, %			SEM^2^	P-values^3^	
	0	2.5	5.0		Linear	Quadratic
Phase I (d 14)						
Total anaerobic bacteria	9.23^b^	9.32^ab^	9.50^a^	0.05	0.020	0.446
*Bifidobacterium* spp.	8.73	8.78	8.79	0.05	0.434	0.706
*Clostridium* spp.	7.21	7.17	7.10	0.07	0.277	0.846
Coliforms	6.98	6.95	6.93	0.05	0.517	0.907
Phase II (d 28)						
Total anaerobic bacteria	9.38^b^	9.46^ab^	9.57^a^	0.16	0.020	0.786
*Bifidobacterium* spp.	8.82	8.85	8.88	0.18	0.379	0.976
*Clostridium* spp.	7.30	7.26	7.24	0.22	0.400	0.887
Coliforms	7.16	7.11	7.08	0.23	0.362	0.814

^ab^Values with different superscripts of the row significantly differ (p < 0.05).

^1^Data represent means based on 6 replicates per treatment.

^2^Standard error of means.

^3^Linear and quadratic effects of increasing citrus pulp and *Bacillus subtilis* fermentation biomass in the diet.

## Discussion

Among several bacterial species used as probiotics, spore-forming *Bacillus* spp. are preferred due to the high resistance of their spores to harsh environments and long-term storability at ambient temperatures [19, 20]. We have reported previously that *B. subtilis* fermentation biomass prepared by solid substrate fermentation with corn-soybean meal or citrus juice waste substrate has potential for improving growth performance, nutrient retention, and intestinal morphology as well as reducing harmful intestinal bacteria in broilers and weanling pigs [11, 14, 18, 21]. In this study, dried citrus pulp and fish by-product was fermented with *B. subtilis*, and the resulting fermentation biomass was supplemented to weanling pig diets.

In the present study, dietary supplementation with 5.0% citrus pulp, fish by-product and *B. subtilis* fermentation biomass improved the feed efficiency of weanling pigs. These results are in agreement with data reported by Lee et al. [14], who observed improved feed efficiency in pigs fed diet supplemented with *B. subtilis* LS 1–2 grown on citrus juice waste. Similarly, a previous study reported that dietary inclusion of *B. subtilis* LS 1–2 and corn-soybean meal or citrus juice waste fermentation biomass can improve the feed conversion efficiency of broilers [11, 21]. In contrast, this study found that citrus pulp, fish by-product, and *B. subtilis* fermentation biomass had no effects on ADG and ADFI of weanling pigs. This discrepancy could be attributed to variations in the administration level of probiotic products, health status within herds, farm hygiene, diet composition, feed forms, and interactions with other dietary feed additives [22, 23]. Other studies also reported improved growth performance in pigs fed diet supplemented with probiotic products containing *Bacillus* spp. [24–27]. The fermentation biomass in the present study was prepared by fermentation of citrus pulp with *B. subtilis*, and the resultant biomass included probiotic microbes as well as secondary metabolites produced during microbial fermentation, as previously described [28, 29].

In this study, dietary supplementation with 5.0% citrus pulp, fish by-product, and *B. subtilis* fermentation biomass improved ATTD of DM, GE, and ash during phase I (d 0–14). Our results confirm the findings of Lee et al. [14], who evaluated the effects of *B. subtilis* fermentation biomass dietary supplementation on weanling pigs and observed improved ATTD of DM and GE during phase I (d 0–14). Similarly, it was reported that weanling pigs fed diet supplemented with probiotic products prepared using *B. subtilis* and corn-soybean meal as a substrate show improved coefficient of total tract digestibility of DM and GE [30]. In contrast to the results of Choi et al. [30], Lee et al. [14], and the present study, Chen et al. [26] and Wang et al. [27] observed no effects of *Bacillus*-based probiotic products on the ATTD of nutrients in growing and finishing pigs. Variations in nutrient digestibility in weanling, growing, and finishing pigs indicate that the age of pigs is a considerable factor affecting the efficacy of *B. subtilis* and its fermentation biomass. In the present study, the ATTD of nutrients during phase II (d 15–28) was not affected by dietary treatments, possibly due to the presence of developed and stable intestinal microflora during phase II. Stavric and Kornegay [31] reported that probiotics are more effective in pigs during microflora development or when microflora stability is impaired.

It has been well established that probiotic products favorably affect the host animal by improving intestinal balance [19], by creating gut micro-ecological conditions that suppress harmful microorganisms, and by favoring beneficial microorganisms [14, 18, 32]. Positive effects of probiotic products containing *B. subtilis* [11, 18, 21, 33] have been reported previously. In the present study, pigs fed diets supplemented with 5.0% citrus pulp, fish by-product, and *B. subtilis* fermentation biomass showed greater total anaerobic bacteria populations by d 14 and 28, whereas there were no effects on *Bifidobacterium* spp., *Clostridium* spp., and coliform populations. In contrast, Lee et al. [14] observed that weanling pigs fed diet supplemented with corn-soybean and *B. subtilis* fermentation biomass show no difference in total anaerobic bacterial populations, whereas cecal *Clostridium* spp. and coliform populations are significantly reduced. This discrepancy might be due to variations in the type and dose of probiotic product, method of probiotic fermentation, and health status of piglets. In this study, we used healthy piglets with no symptoms of diarrhea.

## Conclusions

In conclusion, the results obtained in present study indicate that dietary supplementation with 5.0% citrus pulp, fish by-product, and *B. subtilis* fermentation biomass has the potential to improve feed efficiency, nutrient digestibility, fecal microflora, and cost savings in weanling pigs without affecting growth performance.
